# Anthropometric Measures, Presence of Metabolic Syndrome, and Adherence to Physical Activity Guidelines Among African American Church Members, Dallas, Texas, 2008

**Published:** 2010-12-15

**Authors:** Kerem Shuval, Julie DeVahl, Liyue Tong, Nora Gimpel, Mark J. DeHaven, Jenny J. Lee

**Affiliations:** University of Texas School of Public Health, Division of Epidemiology; University of Texas, Southwestern Medical Center, Dallas, Texas; University of Texas, Southwestern Medical Center, Dallas, Texas; University of Texas, Southwestern Medical Center, Dallas, Texas; University of Texas, Southwestern Medical Center, Dallas, Texas; Georgia State University, Columbus, Georgia

## Abstract

**Introduction:**

The low prevalence of physical activity among African Americans and high risk of cardiovascular disease lends urgency to assessing the association between metabolic syndrome, abdominal obesity, and adherence to current physical activity guidelines. Few studies have examined this association among African American adults.

**Methods:**

We examined the association between demographic characteristics, anthropometric measures, and metabolic syndrome and adherence to the 2008 Department of Health and Human Services guidelines for moderate and vigorous physical activity. Participants were 392 African American church members from congregations in Dallas, Texas. Physical activity levels were assessed via a validated questionnaire (7-Day Physical Activity Recall), and metabolic syndrome was determined on the basis of the American Heart Association/National Heart, Lung, and Blood Institute diagnostic criteria. We used bivariate and multinomial logistic regression to examine the associations.

**Results:**

Meeting guidelines for vigorous physical activity was significantly and independently associated with the absence of metabolic syndrome among women (odds ratio, 4.71; 95% confidence interval, 1.63-13.14; *P* = .003), after adjusting for covariates. No association was found between meeting moderate or vigorous physical activity guidelines and metabolic syndrome among men. Meeting physical activity guidelines was not associated with body mass index or waist circumference among this sample of predominantly overweight and obese African American church members.

**Conclusion:**

Results indicate that meeting the 2008 guidelines for vigorous physical activity is associated with the absence of metabolic syndrome among African American women. This finding might suggest the need to integrate vigorous physical activity into interventions for African American women as a preventive therapy for cardiovascular risk.

## Introduction

The health benefits of physical activity are well established (1). Being physically active decreases the risk for premature death, coronary artery disease, obesity, diabetes, hypertension, cancer, and depression — thereby lowering medical and medication costs and improving quality of life (1). Moreover, a dose-response relationship has been found between the intensity and duration of physical activity and death rates (2). In a prospective cohort study, Byberg et al found that the absolute (all-cause) death rate was 27.1, 23.6, and 18.4 per 1,000 people in groups with low, medium, and high physical activity levels, respectively (2). Both large prospective cohorts and randomized controlled trials have found type 2 diabetes to be preventable by increasing physical activity levels (3,4). Additionally, metabolic syndrome, a clustering of at least 3 risk factors for cardiovascular disease, has been found to be inversely associated with higher levels of physical activity and cardiorespiratory fitness (5).

Despite being aware of the health benefits of physical activity, many people do not adhere to the recommended guidelines for health-promoting activity (6). Only 30% of US adults meet the recommended level of physical activity. White Americans, however, are 1.6 times more likely to meet these recommendations than African Americans (33% vs 21%, respectively). Conversely, African Americans have a higher prevalence of chronic disease, including hypertension, diabetes, and obesity (7). Thus, promoting physical activity in the African American population may reduce these disparities in chronic disease. Ample studies have attempted to promote physical activity through faith-based organizations in the African American community by using community-based participatory research (CBPR) (8,9). Insufficient studies, however, have used a rigorous randomized controlled trial design, and even fewer have relied on objectively measured cardiovascular risk factors (eg, hypertension, diabetes, metabolic syndrome) to assess the effect of interventions. Using CBPR, the GoodNEWS (Genes, Nutrition, Exercise, Wellness, and Spiritual Growth) clinical trial seeks to assess the effect of a health promotion intervention on modifiable cardiovascular risk factors among African American church members.

We used GoodNEWS data to examine the association between African American church members' anthropometric measures, presence of metabolic syndrome, and the US Department of Health and Human Services (HHS) 2008 Physical Activity Guidelines (10). On the basis of these guidelines, for substantial health benefits, adults should engage in at least 150 minutes of moderate-intensity physical activity or at least 75 minutes of vigorous-intensity physical activity per week (10). However, there is insufficient evidence linking anthropometric measures, the presence of metabolic syndrome, and adherence to physical activity guidelines among African American church members; this study attempts to bridge this gap in the literature.

## Methods

### Study design, population, and setting

The GoodNEWs trial was an 18-month extended maintenance study of a sample of adult participants from African American congregations participating in the GoodNEWS faith-based lay health promotion study. Baseline data were collected from September through October 2008, and randomization occurred in December 2008. Congregations were randomized into intervention and control groups. The intervention group involved a health promotion program tailored to the congregation and focusing on wellness principles in 6 dimensions of health (physical, spiritual, social, intellectual, mental, and environmental) with an emphasis on physical activity and a healthful diet, along with continuous staff and group support. The control group was limited to minimal staff contact and support. The primary hypothesis of the trial is that compared with control congregations, at the end of 18 months, participants from intervention congregations would significantly increase their physical activity levels and adherence to a healthful diet. Based on this hypothesis, sample size was calculated to detect a 3% difference in energy expenditure and 15% difference in intake of saturated fat. Thus, 20 sites, each with 20 participants (n = 400), are required to detect an effect size of 0.30 with an intra-class correlation of .015 (power = 0.83) (11).

We approached 20 African American churches from the southern sector of Dallas, Texas, a predominantly low-income area with primarily minority residents. The median annual household income in South Dallas is low ($22,183), and the racial/ethnic makeup is 36% Hispanic and 26% African American (12). Pastors from the churches recruited at least 1 lay health promoter (LHP) to participate in a 4-day (21-hour) curriculum emphasizing lifestyle (physical activity and diet) modification, community resources for health promotion, and methods of establishing a health program in their congregation. Of the 20 churches approached, 18 churches and 20 LHPs completed the required training. These LHPs were asked to recruit at least 20 congregation members who met inclusion criteria (ie, members of the selected congregations and aged 18-70 y) to participate in the study. LHPs signed up 13 to 54 congregation members who met inclusion criteria from each church (584 total). Of the 584 who signed up, congregation members were excluded if they 1) did not show up for measurements (n = 122), 2) did not provide consent (n = 66), or 3) did not complete baseline measurements (n = 4). Thus, 67% of participants who initially expressed interest were included in the study.

We performed cross-sectional analysis of baseline data (September-October 2008) from the GoodNEWS trial among 392 participants from 18 congregations. We examined pertinent measures to assess whether body mass index (BMI), waist circumference, and presence of metabolic syndrome were associated with meeting physical activity guidelines. Participants provided written informed consent prior to completing surveys, anthropometric and blood pressure measurements, and blood sampling. The institutional review board of the University of Texas Southwestern Medical Center approved the study.

### Measures

**Physical activity**

We measured physical activity via a validated questionnaire, the 7-Day Physical Activity Recall (PAR) (13). This tool has been used in numerous studies and settings and has an established reliability and validity (14,15). The PAR provides details on the duration, intensity, frequency, and type of activity during the past 7 days and is representative of typical weekly patterns. The PAR estimates energy expenditure by asking participants to recall the amount of time spent sleeping and in moderate, hard, and very hard activities during the previous 7 days. Time in each category is multiplied by an established metabolic equivalent of task (MET) value. Based on the HHS 2008 Physical Activity Guidelines (10), we created 3 categories for this variable: 1) meeting vigorous physical activity guidelines, at least 75 minutes per week of vigorous-intensity physical activity (≥6 METs); 2) meeting moderate intensity guidelines, at least 150 minutes per week of moderate intensity activity (3-5.9 METs); and 3) meeting neither moderate nor vigorous physical activity guidelines.

**Anthropometric measurements**

We used standard equipment to measure height and weight and classified participants as underweight (<18.5 kg/m^2^), normal weight (18.5-24.9 kg/m^2^), overweight (25.0-29.9 kg/m^2^), obese class 1 (30.0-34.9 kg/m^2^), obese class 2 (35.0-39.9 kg/m^2^), and obese class 3 (≥40.0 kg/m^2^) (16). We used 3 categories for data analysis: normal weight (<25.0 kg/m^2^), overweight (25.0-29.9 kg/m^2^), and obese (≥30.0 kg/m^2^). We used standard equipment and methods to measure waist circumference and defined elevated waist circumference as at least 102 cm in men or at least 88 cm in women (16).

**Blood pressure and laboratory measurements**

We measured blood pressure according to the protocol from the Joint National Committee on Prevention, Detection, Evaluation, and Treatment of High Blood Pressure (JNC 7) and classified blood pressure according to the JNC 7 guidelines (17). Capillary blood was drawn via fingerstick, after a 12-hour fast, for measuring plasma lipids and glucose.

We adhered to the American Heart Association/National Heart, Lung, and Blood Institute diagnostic criteria (18) to classify participants as having metabolic syndrome (yes/no). Meeting at least 3 of the following 5 criteria constitutes diagnosis of metabolic syndrome: 1) elevated waist circumference (≥102 cm in men and ≥88 cm in women), 2) elevated triglycerides (≥150 mg/dL) or on drug treatment for elevated triglycerides, 3) low high-density lipoprotein (HDL) cholesterol (<40 mg/dL in men or <50 mg/dL in women) or on drug treatment for low HDL cholesterol, 4) elevated blood pressure (≥130 mm Hg systolic or ≥85 mm Hg diastolic) or antihypertensive drug treatment in a patient with a history of hypertension, and 5) elevated fasting glucose of at least 100 mg/dL or on drug treatment for elevated glucose.

**Demographic variables**

Demographic variables were age; sex; presence of children younger than 18 years in a household (yes/no); marital status (married/not married); education level (high school or less/more than high school); employment status, dichotomized into yes (part-time or full-time employment) or no (unemployment); self-perceived health status, dichotomized into good (good/excellent) or poor (fair/poor); health insurance coverage in the past 12 months (yes/no); and smoking status (never or current smoker; a third category, former smoker, was not reported by any of the participants, and was omitted).

**Statistical analysis**

We generated descriptive statistics of our study population. We considered participants reporting abnormally high physical activity levels (ie, >16 hours of moderate or vigorous physical activity per day) as outliers and excluded them from analysis (19). Thus, out of 392 participants with complete data, 379 participants were analyzed. To determine whether different intensity levels of physical activity are differentially associated with metabolic syndrome and anthropometric measures, we defined physical activity as a 3-level categorical variable (ie, not meeting guidelines, meeting moderate guidelines, meeting vigorous guidelines) (10). We conducted bivariate and multivariate logistic regression analyses to identify the association between levels of physical activity and demographic, anthropometric, and metabolic syndrome variables. For the bivariate analysis, the Pearson *χ^2^* test was used to assess the relationship between categorical variables and physical activity (meeting moderate guidelines vs not meeting guidelines, meeting vigorous guidelines vs not meeting guidelines). A 2-sample *t* test was used to assess the relationship between age and physical activity.

We used multinomial logistic regression to account for the multiple categories of physical activity (ie, not meeting guidelines, meeting moderate guidelines, meeting vigorous guidelines) (20). Statistical significance was set at P ≤ .05. We constructed 3 multivariate models: model 1, demographic variables; model 2, BMI and waist circumference adjusting for demographic variables; and model 3, metabolic syndrome adjusting for demographic variables. We did not include BMI and waist circumference in model 3 because BMI and metabolic syndrome were highly correlated (*χ* ^2^ = 18.21; *P* ≤ .001), and waist circumference is one of the components of metabolic syndrome. Additionally, we stratified the multivariate models by sex because sex was significantly associated with physical activity in the bivariate analysis. Multivariate regression results are reported as the odds ratios (ORs) and 95% confidence intervals (CIs) of 1) meeting moderate physical activity guidelines relative to participants not meeting the guidelines (ie, referent group) and 2) meeting vigorous physical activity guidelines relative to the referent group. We analyzed data using SAS version 9.2 (SAS Institute, Inc, Cary, North Carolina).

## Results

Participants were African American church members with a mean age of 49 years, mostly women, with more than high school education (74%), and employed either part-time or full-time (71%) (Table 1). Most participants perceived their health to be good or excellent, and most were obese. Additionally, almost half of participants had metabolic syndrome, and most (80%) had elevated waist circumference. Metabolic syndrome was more prevalent among women compared with men (54% and 36%, respectively; *χ* ^2^ = 8.0, *P* < .01), as was elevated waist circumference (84% and 58%, respectively; *χ* ^2 ^= 23.0, *P* < .01).

Bivariate analyses indicate that sex was significantly associated with meeting both moderate and vigorous physical activity guidelines (Table 2). Men were more likely to meet moderate and vigorous physical activity guidelines than were women. Participants' age was significantly associated with meeting vigorous physical activity guidelines only.

In model 1 of the multivariate analysis (Tables 3 and 4), age was inversely associated with meeting vigorous physical activity guidelines among men and women and inversely associated with meeting the moderate guidelines among men only. In model 2, neither BMI nor waist circumference were independently associated with meeting moderate or vigorous physical activity guidelines for men or women after controlling for covariates (Tables 3 and 4). In model 3, meeting vigorous physical activity guidelines was associated with the absence of metabolic syndrome among women after controlling for covariates (Table 4, Figure). No association was found between meeting moderate or vigorous physical activity guidelines and the absence of metabolic syndrome among men.

**Figure 1:**
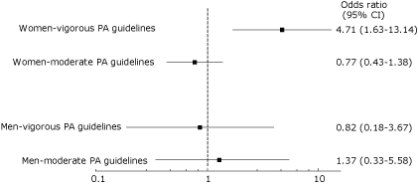
Adjusted odds ratios for meeting physical activity (PA) guidelines according to metabolic syndrome (absence vs presence) among African American church members, Dallas, Texas, 2008. The width of the horizontal line represents the 95% confidence interval of the estimate, and the square represents the point estimate. Values are adjusted for age, education, marital status, smoking, employment, health insurance coverage, perceived health, children younger than 18 years living at home (via multinomial logistic regression), and sex (via stratification).

**Table:** 

**Characteristic**	**Odds Ratio (95% Confidence Interval)**
Women — vigorous PA guidelines	4.71 (1.63-13.14)
Women — moderate PA guidelines	0.77 (0.43-1.38)
Men — vigorous PA guidelines	0.82 (0.18-3.67)
Men — moderate PA guidelines	1.37 (0.33-5.58)

## Discussion

The finding of an association between metabolic syndrome and vigorous but not moderate physical activity is important because it pertains to the intensity of physical activity required to obtain metabolic health benefits among African American church members. The study's findings are consistent with the recent HHS guidelines supporting the health benefits of vigorous physical activity (10). Numerous studies have found an inverse relationship between vigorous physical activity and metabolic syndrome; however, many have also found that moderate-intensity activity is associated with a reduced risk of metabolic syndrome (5,21,22). Incomplete understanding of the relationship between metabolic syndrome and levels of physical activity among African Americans is partially due to scant evidence examining the relationship between these variables among African Americans in any setting. In a cross-sectional study, Irwin et al found an inverse relationship between levels of moderate and vigorous physical activity and presence of metabolic syndrome among a multiethnic middle-aged female population (including African Americans) (5). Additionally, in a large prospective study, Katzmarzyk et al found that physical activity reduced the risk for metabolic syndrome among black and white women and men in Canada (23). Other studies have consistently found an inverse association between physical activity and the lack of metabolic syndrome among middle-aged white men in the United States (24), middle-aged and older men and women in China (22), and adult men and women in Australia (25).

The results of the present study, indicating an association between absence of metabolic syndrome and vigorous physical activity among African American women but not men, contradict the existing literature. The biological mechanism through which physical activity affects metabolic syndrome, for example, via increasing insulin sensitivity and improving endothelial function (5,22,25), should not differentiate among primarily middle-aged men and women. In the present study, the women's sample may be more representative of the true population than the men's sample because of larger sample size — 304 and 75 participants, respectively. Another explanation for the difference in findings could be a difference in physical activity self-reporting among men and women on the 7-Day PAR, which led to misclassification (ie, information bias). However, these 2 explanations are not likely, since the prevalence of physical activity in our sample is similar to findings from large representative national surveys. For example, 41% of men and 34% of women met moderate physical activity guidelines in the present study, compared with 45% and 31% of African American men and women, respectively, in the Behavioral Risk Factor Surveillance System survey from 2005 (26).

We also found that neither BMI nor waist circumference were significantly associated with meeting moderate or vigorous physical activity guidelines. These findings are consistent with a study by Yu et al revealing an association between physical activity and metabolic syndrome, independent of body composition (22). Yet other studies suggest an inverse relationship between abdominal fat and physical activity (27). Ohkawara et al, in a systematic review, found a dose-response effect between an increase in physical activity and decrease in visceral fat (28). Rennie et al similarly found an inverse relationship between physical activity and BMI and waist circumference (21). The fact that the current study did not reveal such an association may stem from the homogeneity of the sample pertaining to participants' anthropometric measures (86% of participants were overweight/obese, and 79% had elevated waist circumference) (27).

The study has limitations. Because the study design is cross-sectional, a temporal and causal relation cannot be determined between participants' anthropometric measures, presence of metabolic syndrome, and meeting physical activity guidelines. Additionally, the results may not be representative of African American church members elsewhere or African Americans elsewhere in general, since the study population consisted of volunteers from a specific geographical area. However, the prevalence of physical activity in this sample was similar to that of the general African American population. In contrast, the prevalence of elevated waist circumference and metabolic syndrome were 1.8 and 2.2 times higher (respectively) in our sample compared with a nationally representative sample of African Americans (29,30). Furthermore, even though we assessed physical activity via a validated and widely used tool (7-Day PAR) (13,14), the tool is based on self-report rather than more objective measures, such as accelerometry.

Nonetheless, our findings contribute to the literature examining the association between physical activity and metabolic syndrome among African Americans. Furthermore, though previous studies have assessed and attempted to promote physical activity among African Americans in faith-based organizations, none have examined metabolic correlates. The study indicates that engaging in recommended levels of vigorous physical activity is associated with the absence of metabolic syndrome in a predominantly overweight/obese sample of African American women. Because of the disproportionate prevalence of metabolic syndrome among African American women (30), the study might suggest the need to introduce vigorous physical activity as a preventive therapy. Program planners should examine health promotion strategies to promote vigorous physical activity in this study population. This recommendation, based on cross-sectional data, needs to be substantiated in a prospective study with a more representative sample of African Americans.

## Figures and Tables

**Table 1:** Characteristics of 379 African American Church Members, Dallas, Texas, 2008^a^

Characteristic	**No. (%)**
**Education**	
≤High school graduate	98 (26)
>High school graduate	275 (74)
**Age, mean (SD), y**	49 (12)
**Married**	
No	183 (49)
Yes	193 (51)
**Employed**	
No	110 (29)
Yes	263 (71)
**Smoking status**	
Never	276 (81)
Current	66 (19)
**Sex**	
Men	75 (20)
Women	304 (80)
**Perceived health**	
Poor	73 (20)
Good	297 (80)
**Health insurance coverage, past 12 months**	
No	60 (16)
Yes	319 (84)
**Children <18 y living at home**	
No	233 (61)
Yes	146 (39)
**BMI^b^, kg/m^2^**	
Underweight	2 (1)
Normal weight	51 (13)
Overweight	71 (19)
Obese 1	96 (25)
Obese 2	75 (20)
Obese 3	84 (22)
**Elevated waist circumference^c^**	
No	74 (20)
Yes	305 (80)
**Metabolic syndrome^d^**	
No	196 (52)
Yes	183 (48)

Abbreviation: BMI, body mass index.

a Categories of variables do not add up to n = 379 because of missing responses.

b The following classifications (16) were used: underweight, BMI <18.5 kg/m^2^; normal weight, BMI 18.5-24.9 kg/m^2^; overweight, BMI 25-29.9 kg/m^2^; obese 1, BMI 30.0-34.9 kg/m^2^; obese 2, BMI 35.0-39.9 kg/m^2^; and obese 3, BMI ≥40.0 kg/m^2^.

c Defined as elevated when ≥102 cm in men or ≥88 cm in women (16).

d Defined as having ≥3 of the 5 diagnostic criteria based on American Heart Association/National Heart, Lung, and Blood Institute scientific statement (18).

**Table 2:** Prevalence of Meeting Physical Activity Guidelines by Demographic Characteristics, Anthropometric Measures, and Presence of Metabolic Syndrome Among 379 African American Church Members, Dallas, Texas, 2008^a^

**Characteristic**	Meeting Moderate PA Guidelines, No. (%)	*P* Value^b^	Meeting Vigorous PA Guidelines, No. (%)	*P* Value^b^
**Education**				
≤High school graduate	32 (33)	.70	17 (17)	.87
>High school graduate	97 (35)		44 (16)	
**Age, mean (SD), y**	50 (12)	.95	43 (13)	.001
**Married**				
No	60 (33)	.43	28 (15)	.48
Yes	69 (36)		33 (17)	
**Employed**				
No	88 (33)	.55	46 (17)	.50
Yes	42 (38)		15 (14)	
**Smoking status**				
Never	98 (36)	.82	43 (16)	.34
Current	21 (32)		14 (21)	
**Sex**				
Men	29 (39)	.04	21 (28)	.001
Women	102 (34)		40 (13)	
**Perceived health**				
Poor	24 (33)	.74	13 (18)	.78
Good	106 (36)		47 (16)	
**Health insurance coverage in past 12 months**				
No	16 (27)	.34	14 (23)	.22
Yes	115 (36)		47 (15)	
**Children <18 y living at home**				
No	80 (34)	.86	37 (16)	.85
Yes	51 (35)		24 (16)	
**BMI^c^, kg/m^2^**				
Normal weight	17 (32)	.70	8 (15)	.49
Overweight	24 (33)		9 (13)	
Obese	90 (35)		44 (17)	
**Elevated waist circumference^d^**				
No	26 (33)	.74	14 (18)	.80
Yes	105 (35)		47 (16)	
**Metabolic syndrome^e^**				
No	64 (34)	.90	33 (18)	.42
Yes	67 (35)		28 (15)	

Abbreviation: PA, physical activity; BMI, body mass index.

a Some categories of variables do not total 379 because of missing responses.

b Indicates significance compared with not meeting 2008 guidelines, defined by the US Department of Health and Human Services (10).

c Three BMI categories were used: 1) normal weight, BMI <25 kg/m^2^; 2) overweight, BMI, 25.0-29.9 kg/m^2^; and 3) obese, BMI ≥30.0 kg/m^2^.

d Defined as ≥102 cm in men or ≥88 cm in women (16).

e Defined as having ≥3 of the 5 diagnostic criteria based on American Heart Association/National Heart, Lung, and Blood Institute Scientific Statement (18).

**Table 3:** Odds of Meeting Physical Activity Guidelines by Demographic Characteristics, Anthropometric Measures, and Presence of Metabolic Syndrome Among 379 Male African American Church Members, Dallas, Texas, 2008^a^

Characteristic	Model 1		Model 2		Model 3	

	Moderate PA, OR (95% CI)	Vigorous PA, OR (95% CI)	Moderate PA, OR (95% CI)	Vigorous PA, OR (95% CI)	Moderate PA, OR (95% CI)	Vigorous PA, OR (95% CI)
**Education**						
≤High school graduate	1 [Reference]					
>High school graduate	0.53 (0.12- 2.29)	1.63 (0.33-8.06)	0.43 (0.09-2.01)	1.48 (0.28-7.73)	0.51 (0.12-2.20)	1.73 (0.34-8.59)
**Age, mean (SD), y**	0.89 (0.82-0.97)^b^	0.90 (0.83-0.99)^b^	0.90 (0.82-0.99)^b^	0.90 (0.82-0.99)^b^	0.90 (0.82-0.98)^b^	0.91 (0.83-1.00)
**Married**						
No	1 [Reference]					
Yes	10.25 (1.45-72.5)^b^	2.57 (0.41-16.00)	8.15 (0.96-68.61)	3.43 (0.43- 27.04)	9.50 (1.38-65.39)^b^	2.82 (0.44-17.87)
**Employed**						
No	1 [Reference]					
Yes	1.11 (0.58-2.12)	0.75 (0.30-1.88)	1.02 (0.54-1.93)	0.80 (0.32-2.00)	1.11 (0.58-2.12)	0.75 (0.30-1.92)
**Smoking**						
Never	1 [Reference]					
Current	0.22 (0.07-1.28)	0.67 (0.16-2.88)	0.24 (0.05-1.15)	0.54 (0.11-2.46)	0.29 (0.06-1.27)	0.59 (0.13-2.61)
**Perceived health**						
Poor	1 [Reference]					
Good	1.17 (0.18-7.55)	0.53 (0.08-3.28)	0.98 (0.13-6.50)	0.51 (0.07-3.42)	1.00 (0.13-7.58)	0.48 (0.06-3.62)
**Health insurance coverage, past 12 months**						
No	1 [Reference]					
Yes	3.09 (0.37-25.30)	0.77 (0.10-5.9)	3.94 (0.45-34.09)	0.74 (0.09- 6.03)	3.03 (0.37-24.79)	0.80 (0.10-6.21)
**Children <18 y living at home **						
No	1 [Reference]					
Yes	0.22 (0.04-1.11)	0.49 (0.08-2.92)	0.17 (0.02-1.07)	0.37 (0.05-2.69)	0.22 (0.04-1.11)	0.48 (0.08-2.76)
**BMI^c^, kg/m^2 ^**						
Normal weight	NC	NC	1 [Reference]		NC	NC
Overweight	NC	NC	0.31 (0.02-3.89)	0.23 (0.02-2.50)	NC	NC
Obese	NC	NC	0.66 (0.04-10.03)	0.64 (0.05-7.58)	NC	NC
**Elevated waist circumference^d^**						
No	NC	NC	1 [Reference]		NC	NC
Yes	NC	NC	2.64 (0.34-20.13)	0.83 (0.10-6.89)	NC	NC
**Metabolic syndrome^e^**						
No	NC	NC	NC	NC	1.37 (0.33-5.58)	0.82 (0.18-3.67)
Yes	NC	NC	NC	NC	1 [Reference]	

Abbreviations: PA, physical activity; OR, odds ratio; CI, confidence interval; BMI, body mass index; NC, not calculated.

a Vigorous and moderate physical activity guidelines (defined by the US Department of Health and Human Services [10]) are separately compared with the reference category of not meeting physical activity guidelines. Model 1 accounted for demographic characteristics, model 2 for BMI and waist circumference adjusting for demographic characteristics, and model 3 for metabolic syndrome adjusting for demographic characteristics.

b *P* < .05.

c Three BMI categories were used: 1) normal weight, BMI <25.0 kg/m^2^; 2) overweight, BMI 25.0-29.9 kg/m^2^; and 3) obese, BMI ≥30.0 kg/m^2^.

d Defined as ≥102 cm in men or ≥88 cm in women (16).

e Defined as having ≥3 of the 5 diagnostic criteria based on American Heart Association/National Heart, Lung, and Blood Institute scientific statement (18).

**Table 4:** Odds of Meeting Physical Activity Guidelines by Demographic Characteristics, Anthropometric Measures, and Presence of Metabolic Syndrome Among 379 Female African American Church Members, Dallas, Texas, 2008^a^

Characteristic	Model 1		Model 2		Model 3	

	Moderate PA, OR (95% CI)	Vigorous PA, OR (95% CI)	Moderate PA, OR (95% CI)	Vigorous PA, OR (95% CI)	Moderate PA, OR (95% CI)	Vigorous PA, OR (95% CI)
**Education**						
≤High school graduate	1 [Reference]					
>High school graduate	1.38 (0.68-2.78)	0.72 (0.29-1.77)	1.30 (0.65-2.60)	0.81 (0.33-1.99)	1.45 (0.71-2.97)	0.56 (0.21-1.45)
**Age, mean (SD), y**	1.01 (0.98-1.03)	0.93 (0.89-0.97)^b^	1.01 (0.98-1.04)	0.94 (0.91-0.98)	1.01 (0.98-1.04)	0.95 (0.91-0.98)
**Married**						
No	1 [Reference]					
Yes	0.82 (0.47-1.44)	1.22 (0.54-2.76)	0.80 (0.46-1.41)	1.11 (0.49-2.54)	0.82 (0.47-1.43)	1.27 (0.55-2.94)
**Employed**						
No	1 [Reference]					
Yes	1.11 (0.58-2.12)	0.75 (0.30-1.88)	1.02 (0.54-1.93)	0.80 (0.32-2.00)	1.11 (0.58-2.12)	0.75 (0.30-1.92)
**Smoking **						
Never	1 [Reference]					
Current	1.08 (0.51-2.27)	2.07 (0.73-5.87)	1.04 (0.49-2.20)	1.89 (0.66-5.37)	1.05 (0.50-2.22)	2.38 (0.80-7.07)
**Perceived health**						
Poor	1 [Reference]					
Good	1.25 (0.62-2.51)	2.11 (0.69-6.42)	1.33 (0.66-2.69)	2.41 (0.78-7.36)	1.30 (0.64-2.63)	1.48 (0.47-4.70)
**Health insurance coverage, past 12 months**						
No	1 [Reference]					
Yes	1.33 (0.56-3.16)	1.00 (0.34-2.89)	1.25 (0.54-2.90)	1.01 (0.35-2.92)	1.27 (0.53-3.03)	1.22 (0.41-3.68)
**Children <18 y living at home**						
No	1 [Reference]					
Yes	1.22 (0.65-2.29)	0.44 (0.18-1.08)	1.24 (0.66-2.32)	0.46 (0.19-1.11)	1.22 (0.65-2.28)	0.47 (0.19-1.18)
**BMI^c^, kg/m^2 ^**						
Normal weight	NC	NC	1 [Reference]		NC	NC
Overweight	NC	NC	1.37 (0.49-3.82)	0.76 (0.14-4.15)	NC	NC
Obese	NC	NC	1.51 (0.53-4.29)	0.80 (0.17-3.71)	NC	NC
**Elevated waist circumference^d^**						
No	NC	NC	1 [Reference]		NC	NC
Yes	NC	NC	0.95 (0.37-2.42)	6.89 (0.72-65.93)	NC	NC
**Metabolic syndrome^e^**						
No	NC	NC	NC	NC	0.77 (0.43-1.38)	4.71 (1.63-13.14)^b^
Yes	NC	NC	NC	NC	1 [Reference]	

Abbreviations: PA, physical activity; OR, odds ratio; CI, confidence interval; body mass index, BMI; NC, not calculated.

a Vigorous and moderate physical activity guidelines (defined by the US Department of Health and Human Services [10]) are separately compared with the reference category of not meeting physical activity guidelines. Model 1 accounted for demographic characteristics, model 2 for BMI and waist circumference adjusting for demographic characteristics, and model 3 for metabolic syndrome adjusting for demographic characteristics.

b *P* < .05.

c Three BMI categories were used: 1) normal weight, BMI <25.0 kg/m^2^; 2) overweight, BMI 25.0-29.9 kg/m^2^; and 3) obese, BMI ≥30.0 kg/m^2^.

d Defined as ≥102 cm in men or ≥88 cm in women (16).

e Defined as having ≥3 of the 5 diagnostic criteria based on American Heart Association/National Heart, Lung, and Blood Institute Scientific Statement (18).
